# A Novel Plasma-Enhanced Solvolysis as Alternative for Recycling Composites

**DOI:** 10.3390/polym16192836

**Published:** 2024-10-07

**Authors:** Dimitrios Marinis, Dionysios Markatos, Ergina Farsari, Eleftherios Amanatides, Dimitrios Mataras, Spiros Pantelakis

**Affiliations:** 1 Department of Chemical Engineering, University of Patras, GR26504 Patras, Greece; marinis@chemeng.upatras.gr (D.M.); efarsari@chemeng.upatras.gr (E.F.); dim@chemeng.upatras.gr (D.M.); 2 Department of Mechanical Engineering and Aeronautics, University of Patras, GR26504 Patras, Greece; dmark@upatras.gr (D.M.); pantelak@upatras.gr (S.P.)

**Keywords:** recycling, composite, CFRP, CFRC, carbon fibers, plasma in liquids, sustainability

## Abstract

In this work, a plasma-assisted solvolysis method is proposed as an alternative method for the oxidative degradation of carbon fiber-reinforced composites (CFRCs). Nitrogen plasma ignition within bubbles in a concentrated nitric acid solution is employed, combining the synergistic effects of traditional nitric acid solvolysis and plasma chemistry. A comprehensive process flowchart, including steps such as composite pretreatment, matrix dissolution, fiber recovery and cleaning, solvent regeneration and reuse, and waste treatment, is also discussed, highlighting their importance in process effectiveness. Moreover, a study on the effect of the composite’s mass on the plasma-enhanced solvolysis process is conducted, and the results are exploited for the calculation of critical parameters such as efficiency, recovery rates, capacity, fibers quality, energy consumption, consumption of raw materials, operational and installation costs, and environmental impact. A preliminary comparison to other recycling methods based on the literature findings is also attempted, and preliminary metrics to assess the sustainability of the recycling process are proposed.

## 1. Introduction

Due to their unique chemical and mechanical properties, carbon fiber-reinforced composites (CFRCs) are widely used in demanding structural applications such as wind turbine blades manufacturing, the aerospace sector, and the automotive industry. The significant demand for CFRCs is projected to soar to 190 kilotons by 2050 [1,2,3]. Yet, their recycling at their end of life poses a significant challenge due to the resin’s cross-linked nature after curing and shaping. Traditional disposal methods involve either landfilling or incineration, both of which lead to severe environmental contamination and the loss of valuable raw materials. As a result, these methods have been gradually banned in many countries and regions, prompting the development of alternative treatment methods [4,5,6].

Current methods for recovering carbon fibers from waste composites include mechanical, thermal, and chemical processes [7,8,9,10,11,12,13,14,15,16,17,18]. Mechanical methods involve crushing, shredding, or milling, with the resulting material often used as fillers or reinforcements to enhance the mechanical properties of other materials. However, this approach significantly damages the recovered fibers, making it challenging to salvage long fibers [8]. On the other hand, chemical and thermal methods focus on fully recovering the fibers while eliminating the polymer matrix. The thermal method, known as pyrolysis, involves decomposing the organic matrix of the samples in a chemically inert atmosphere through heating [9,10]. Thermal recycling can yield clean fibers while utilizing the resin as fuel. However, thermal processes are typically energy-intensive and costly, demanding temperatures above 400 °C and releasing harmful gases such as CO_2_ and CO [9,10].

Moreover, chemical recycling methods have attracted particular attention because high-value-added fibers and monomers can be recovered through the selective cleavage of chemical bonds in solvent–catalyst systems. More specifically, chemical recycling, also known as solvolysis, separates the resin matrix from CFRCs by breaking the critical chemical bonds in the resin matrix with specific solvents [11]. Compared to other recycling methods, solvolysis is more efficient in degrading composites under mild reaction conditions while causing less damage to the fibers, making it one of the most promising technologies [11].

Three primary solvolysis methods have been proposed: decomposition using super-/sub-critical fluids, alcoholysis, and various wet oxidation techniques. Super-/sub-critical fluids, such as water or organic solvents, offer unique properties that enhance resin decomposition, enabling high-quality fiber recovery [11,12,13]. However, the high pressure (>5 MPa) and temperature (>300 °C) requirements make scaling up challenging and energy-intensive [9]. Alcoholysis relies on solvolysis using organic solvents under alkaline catalysis (such as PEG-NaOH or K_3_PO_4_-ethanol). The lipophilic nature of epoxy resins enhances their breakdown efficiency in organic solvents, primarily alcohols, under catalysis. Alcoholysis operates efficiently under intermediate conditions (100–200 °C and 1–5 MPa), though it faces drawbacks such as the use of high-boiling-point solvents, which might negatively impact the decomposition time [14,15,16].

Wet oxidation methods, utilizing concentrated acidic solutions such as nitric acid and hydrogen peroxide at relatively low temperatures and atmospheric pressure are the most reliable for decomposing unknown types of epoxy CFRCs [17,18,19,20,21]. Nitric acid emerges as the most effective oxidant, ensuring a fiber recovery process that retains up to 98% of the original strength. The decomposition of epoxy resins in nitric acid depends on the cross-linked network between the resin and the hardener of the composite. Amino-cured epoxy resins decompose due to the cleavage of the C-N bonds, while anhydrite-cured resins decompose due to cleavage of the C-O bonds, both via hydrolysis [19,20,21]. In both cases, cleavage of the C-C bonds is the secondary reaction. Eventually, when enough bonds are cleaved, parts of the network start to detach, and the decomposed products maintain structural similarity to the initial resin due to the preservation of the main chains. Consequently, the post-treatment mixture comprises nitrated compounds and residual nitric acid [20,21]. Despite the process efficacy, its drawback lies in the extended decomposition times ranging from 20 to 100 h, raising environmental concerns due to the production of nitrogen oxides and liquid wastes [20].

Recent work has shown that plasma-assisted solvolysis can accelerate the oxidative degradation of CFRCs [22]. The methodology integrates nitrogen plasma into a concentrated nitric acid solution, combining the synergistic effects of traditional nitric solvolysis and plasma chemistry. In the absence of plasma, the reactive species contributing to resin decomposition are limited to NO_2_^+^, H_3_O^+^, NO_3_^−^, and OH. However, when plasma is initiated in a nitric acid solution, several new reactive species are generated (including NO_2_^+^, H_3_O^+^, OH•, H^+^, H•, NO_2_•, H_2_O_2_, O^+^, HO^−^, H_2_, O_2_, O_3_, NO_3_^−^, O_2_^−^, NO_2_^−^, etc.), potentially enhancing resin degradation [22,23,24]. Additionally, as gas diffuses in the liquid phase, plasma is created in the bubbles, which explode near or on the surface of the material, accelerating species diffusion and resin breakdown.

In the current work, the effect of CFRC mass on the plasma-assisted solvolysis process is investigated using a lab-scale reactor, and the results are used to derive key process parameters, such as efficiency, recovery rates, capacity, fiber quality, energy consumption, raw material usage, operational and installation costs, and environmental impact in terms of Global Warning Potential (GWP). A complete process flowchart, detailing steps such as composite pretreatment and dissolution, fiber recovery and cleaning, solvent regeneration, and the re-use and treatment of wastes, is also presented and discussed in relation to their importance for process sustainability.

In addition, a preliminary comparison to other recycling methods based on the literature findings is performed, and the advantages and drawbacks of the process are discussed. Finally, proposed metrics for evaluating the sustainability of the recycling process are also introduced and discussed.

## 2. Materials and Methods

**Figure 1:**
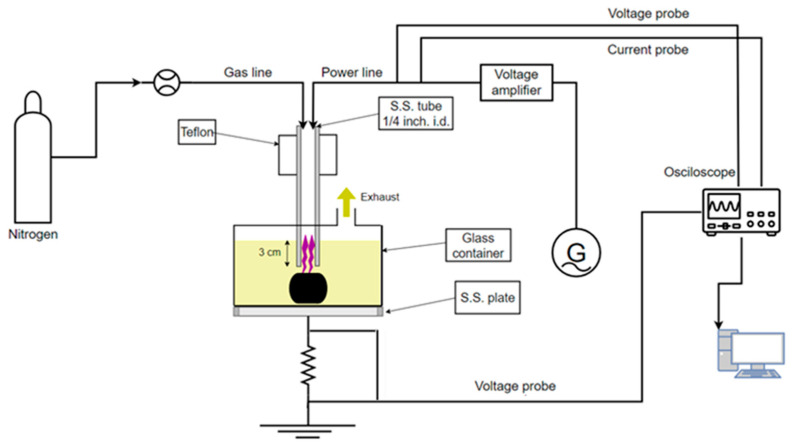
Plasma reactor setup.

Figure 1 illustrates the plasma reactor setup for the plasma-enhanced chemical solvolysis of the CFRCs. The reactor consists of a 2 L glass container where CFRCs with a maximum mass of 1 kg and concentrated (65% wt/wt) HNO_3_ can be placed. The glass vessel is mechanically attached to a stainless-steel plate which acts as the grounded electrode of the reactor. The powered electrode is a stainless-steel tube which also functions as the gas inlet. The electrode is powered by a high-frequency generator (30 kHz signal generator IGBT143, Martignoni Elettrotecnica, Milano, Italy) through a voltage amplifier (IGBT163, Martignoni Elettrotecnica) and is immersed 3 cm into the nitric acid solution, allowing the flowing gas (N_2_ > 99.9%, Novogas) to produce bubbles in the solution.

When the generator is turned on, the plasma is ignited, and the produced active species are dispersed in the reactor, enhancing the degradation of the polymeric matrix. The high voltage applied to the stainless-steel tube is measured using a high-voltage 1000:1 passive probe (P6015A, Tektronix, Beaverton, OR, USA), while the current flow through the setup is monitored by measuring the voltage drop (100:1 passive voltage probe, Hameg HZ53, Mainhausen, Germany) across a 6.5 Ω resistance attached between the stainless-steel bottom plate and the ground. The measurements of the applied voltage and current allow us to calculate the process power [25], which is approximately ~250 W in all experiments. The HNO_3_ volume and the N_2_ flow rate were set at 1.2 L and 4 Lmin^−1^, respectively, in all cases. This combination of plasma power, HNO_3_ volume, and gas flow rate leads to a solution temperature of ~80 °C and is reached about 10 min after plasma ignition.

**Figure 2:**
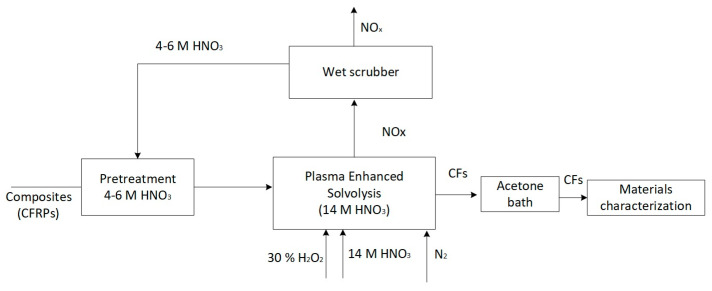
Process flowchart.

In addition to the plasma process, the recovery of CFs from CFRCs requires several other steps that serve either the quality of the final product or the process sustainability. Namely, CFRC pretreatment, liquid waste regeneration, flue gas scrubbing, and rCF cleaning are implemented alongside the plasma process. Figure 2 outlines the entire process flowchart. Initially, the composites are pretreated in a 4 M HNO_3_ solution for CFRC swelling before entering the plasma reactor, where they are treated until total fiber detachment occurs. During treatment, the flue gas is directed into a wet scrubber containing low-concentration HNO_3_ and H_2_O_2_ solution, where the emitted gas mixture is partially converted to HNO_3_, increasing the concentration of the scrubber solution. When the concentration of the scrubbing liquid reaches 4 to 6 M, it is collected and used as the pretreatment liquid. The liquid waste from the plasma reactor is regenerated by adding small amounts of H_2_O_2_ after plasma treatment and the complete dissolution of the composites. The regenerated solution is mixed with fresh HNO_3_ and, in some cases, with the scrubbing liquid until it reaches the desired volume, after which it is used for the next plasma operation cycle. Finally, the fibers are mechanically collected, and if necessary, washed with acetone. Typically, 1 L of acetone is sufficient for cleaning about 1 kg CFs.

Τhe retrieved CFs are characterized by means of SEM-EDX (JEOL 6300, Zhubei City, Taiwan) analysis, and their mechanical properties are measured (Miniature Materials Tester Minimat 2000) according to [26].

For a comprehensive sustainability assessment of the process and, in general, of recycling processes, a framework is needed to provide a holistic perspective. Achieving sustainability in recycling is not just about meeting one criterion but about finding the optimal trade-off between different factors. This need is more obvious in real-world scenarios where the strengthening of one aspect can negatively impact another.

To determine the sustainability of recycling processes, key sustainability pillars are proposed, each representing a critical dimension of the recycling process. These pillars are fundamental to assess and achieve sustainable recycling:

Quality and Process Efficiency Aspects: This pillar includes the mechanical properties of the recycled fibers, focusing on tensile properties such as Young’s modulus and tensile strength, physical properties like the cleanliness of the fibers, recycling processing time (i.e., the time required to obtain the recycled fibers), and recycling efficiency (i.e., the percentage of material fiber mass successfully recycled compared to the fiber mass initially input into the recycling process).

Environmental Aspects: This pillar addresses environmental emissions from the recycling processes, with a focus on greenhouse gases (GHGs), mainly CO_2_ emissions, while also considering other pollutants such as nitrogen oxides (NOx) and volatile organic compounds (VOCs) that may be produced during recycling.

Cost Aspects: This pillar deals with operational costs, encompassing the direct economic expenses associated with the recycling process. These include labor, chemicals, materials, and energy costs, as well as expenses related to infrastructure and potential technology scale-up costs.

Circular Economy Aspects: This pillar focuses on circular economy aspects related to the recycling process, such as the reclamation of resins or the recirculation of chemicals within subsequent recycling loops.

Human Health and Social Impact Aspects: This pillar can evaluate potential health and safety concerns (e.g., hazard levels) associated with the recycling process, as well as metrics related to the social impact of the material sourcing locations (e.g., the country of origin).

In order to quantify all these different pillars and metrics, integrate them into the decision-making process, and extract the final ranking of the processes in terms of their sustainability, an approach centered around Multi-Criteria Decision-Making (MCDM) was proposed by the authors in [27]. However, since the plasma solvolysis process is currently under optimization and a full Life Cycle Assessment (LCA), Life Cycle Costing (LCC), and Social Life Cycle Assessment (SLCA) are still pending, the evaluation remains as a topic for future work. This will be conducted in accordance with the previously described metrics.

The following section defines and presents preliminary process indicators and metrics related to the sustainability of the process.

## 3. Results

In this work, plasma-assisted chemical recycling of CFRCs was tested using custom-made tubes that were supplied by B&T Composites. The materials were manufactured via filament winding of 24 m of continuous 24 K carbon fibers (Tenax^®^-E STS40 E23 24 K 1600tex, Baltimore, MA, USA), while EPIKOTE™ Resin 828 and EPIKURE™ Curing Agent 866 (anhydrite agent) were used for the matrix preparation. The volume and the mass of each tube were approximately 2 cm^3^ and 0.035 kg, respectively, while the nominal mass content of the fibers was 64 ± 3%. To evaluate the process, several experiments were performed by increasing the composites’ masses in the range of 0.035–0.164 kg, while keeping the HNO_3_ volume constant at 1.2 L. A treatment of 0.035 kg of composites in warm HΝO_3_ (~100 °C) was also performed to distinguish the plasma effect from the pure HΝO_3_ effect. In all cases, the treatment was extended up to the complete detachment of the fibers (>99% mass retrieval). Table 1 summarizes the initial mass of the CFRCs, the total mass of the recovered CFs, the total HNO_3_ volume losses in each run, the time required for the complete recovery of the carbon fibers, and the energy required for the complete dissolution of the CFRCs, as calculated from the plasma power (250 W) and the process time. In the absence of plasma, the energy was calculated from the power required to heat the 1.2 L HNO_3_ solution to 100 °C in 10 min and the power required to maintain this temperature for 18 h.

In the presence of plasma, the retrieval of the carbon fibers was accomplished in less than 5.5 h, regardless of the initial mass of the composites. Without plasma, an 18 h treatment was necessary to receive the fibers when warm HNO_3_ was used for the solvolysis of a 0.035 kg composite. The significant decrease in the process time underlines the beneficial role of plasma in CFRCs’ solvolysis. The enhancement of matrix dissolution is mainly attributed to the plethora of active and strong oxidative species generated when plasma is ignited in the HNO_3_ solution. The enhanced diffusivity of species in the CFRC matrix, due to the strong shockwaves that are produced from plasma in liquids, supports the faster dissolution. Additionally, the better distribution of the active species due to high gas flow rate and the plasma-induced temperature increase can further promote the matrix dissolution.

As for the total mass of the recovered fibers, a reasonable linear increase is observed with the CFRCs’ initial mass (Table 1). In fact, the ratio m_rCF_/m_o,CFRC_ is, in all cases, approximately 0.65, which is very close to the expected initial CF mass fraction in the CFRCs. Thus, there are no significant losses of fibers during the plasma treatment and post-treatment, and almost 100% recovery of the CFs from the CFRCs can be assumed.

**Figure 3:**
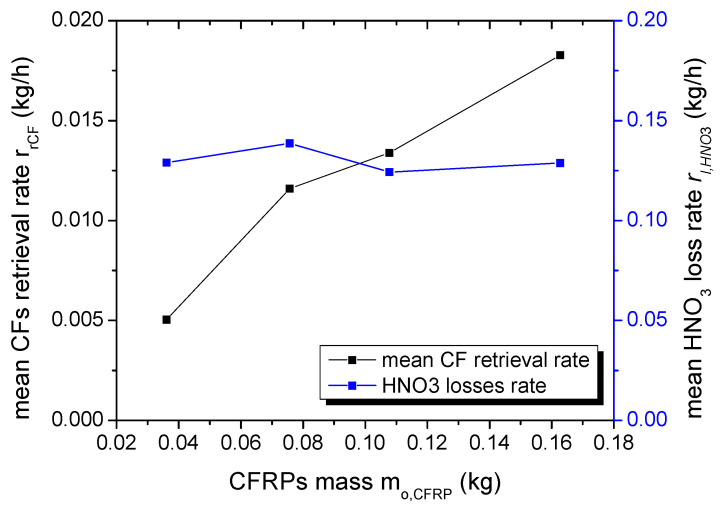
Mean CF retrieval rate r_rCF_ (kg/h, left axis) and HNO_3_ loss rate r_l,HNO3_ (kg/h, right axis) as a function of the initial mass of the composites.

The process time required for the complete dissolution of CFRCs only slightly increases with the CFRCs’ initial mass (Table 1). More precisely, when the mass of the composites was quadrupled, only a 20% increase in process time was observed. This suggests that, in the range of the examined conditions, the oxidizing species produced in the process are in excess, and the initial mass of the composites determines the retrieval rate of the carbon fibers. This is better illustrated in Figure 3 (left axis) where the mean carbon fiber retrieval rate is presented as a function of the initial mass of the composites. The mean CF retrieval rate r_rCF_ was calculated from the total mass of the CFs (m_rCF_, Table 1) that were collected after plasma treatment, cleaned with acetone, dried and weighed, divided by the total process time t. It is also worth noting that the ratio of the CF retrieval rate to the initial composite mass r_rCF_/m_o,CFRC_, which actually defines the hourly process efficiency, remained almost constant for all plasma experiments (0.13 ± 0.02 (kg CFs)/(kg CFRC · h)), i.e., about 13% of the mass of the composite will be recovered as CFs every hour, which corresponds to 20% of the initial fiber mass.

In addition, the HNO_3_ loss rate r_l,HNO3_ (right axis, Figure 3) remained constant (~0.130 ± 0.006 kg/h), which in turn indicates that although HNO_3_ is necessary to dissolve the polymeric matrix, its consumption is not determined by the solvolysis reaction. The observed HNO_3_ losses are more likely dependent on the HNO_3_ solution evaporation caused by the temperature increase, which is induced by the plasma power and the N_2_ flow rate (bubbling). The mean HNO_3_ loss rate r_l,HNO3_ was estimated by subtracting the liquid volume at the end of plasma process from the initial HNO_3_ volume (1.2 L) to calculate the HNO_3_ volume loss (V_l,HNO3_, Table 1), which is then transformed to mass (65% wt/wt HNO_3_ with density ρ = 1.42 kg/L) and divided by the total process time.

**Figure 4:**
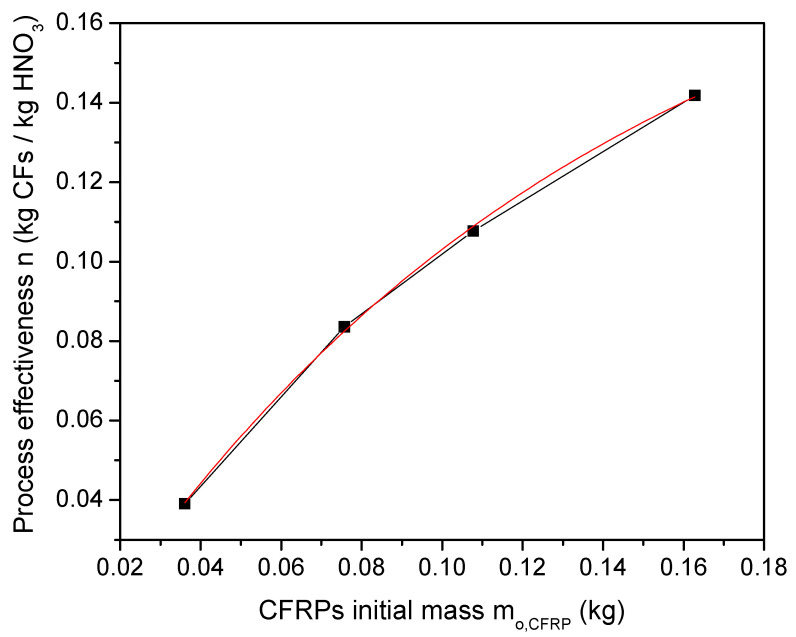
Process effectiveness n as a function of initial mass of the CFRCs.

From the description of the process flow chart (experimental section) and the results presented above, the CF retrieval rate r_rCF_ and the HNO_3_ loss rate r_l,HNO3_ are the most important and key factors for the specific process. The CF retrieval rate r_rCF_ determines the overall productivity and process capacity, while HNO_3_ is the main chemical of the process, and extensive losses will seriously affect operational costs and the process sustainability. In fact, full process optimization requires an optimization of the ratio n = r_rCF_/r_l,HNO3_. This ratio reflects the mass of recovered CFs per mass of HNO_3_ lost; thus, higher values indicate more effective treatment conditions. Figure 4 presents the value of the ratio n as a function of the initial mass of CFRCs, and as one can observe, the increase in the reactor load with CFRCs significantly improves the process effectiveness. An exponential fit of the experimental data results in a relation of the form n = 0.203–0.216e^−7.71mo,CFRC^, which is also included as a red line in Figure 4.

The increase in n is due to the continuous increase in r_rCF_, while r_l,HNO3_ remains almost constant with the CFRCs’ initial mass. The enhancement of r_rCF_ was attributed to an excess of active species and the determination of the retrieval rate from the initial mass of the composites. On the other hand, r_l,HNO3_ depends on the plasma power, N_2_ gas flow, and HNO_3_ initial volume but not on the CFRCs’ initial mass; thus, it remains constant for this set of experiments. Small variations are due to the different process times required for the full decomposition of the CFRCs.

The results for the effectiveness n are far from being optimum, as almost all other process parameters (plasma power, plasma configuration and electrodes, reactor materials, gas flow, HNO_3_ volume, and HNO_3_ concentration) have a strong effect on n. However, they can be used for the extrapolation of experimental data for the retrieval of 1 kg CFs and for the initial studies of process viability and sustainability.

**Figure 5:**
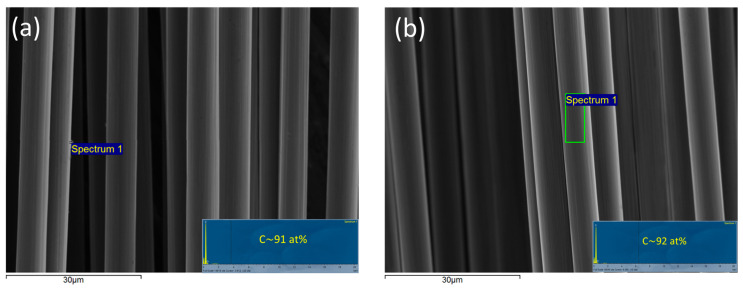
SEM-EDX images: (**a**) rCFs and (**b**) virgin carbon fibers.

Another important parameter of significant impact in process sustainability is the quality of the recycled carbon fibers (rCFs). The quality of plasma treatment and post-cleaning with acetone was monitored by means of SEM–EDX analysis, and the results were compared to those of virgin carbon fibers (vCFs) used for the manufacturing of CFRCs. Figure 5 illustrates representative results, and as one can observe, the surface of the rCFs is undamaged, and no resin residuals can be detected by EDX. The mean diameter of the rCF is calculated ~7 μm which is equal to the diameter of the virgin CFs. Finally, no significant difference in the carbon atomic content of rCF compared to the virgins’ is observed.

**Figure 6:**
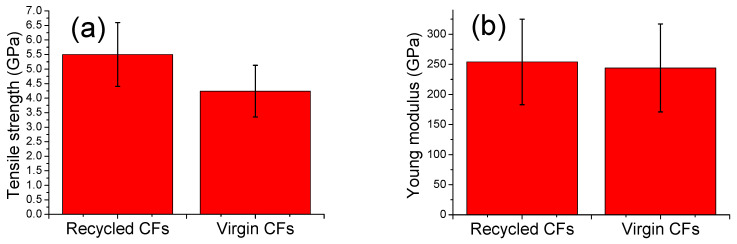
Mechanical properties of the rCFs and the vCFs: (**a**) the normalized single-fiber tensile strength and (**b**) the normalized Young’s modulus.

Moreover, single-fiber mechanical tests took place according to ASTM C1557–14 [26], and the results are presented in Figure 6. The rCFs exhibited an approximately 35% higher single-fiber tensile strength and 5% higher Young’s modulus compared to those of the vCFs. In general, nitric acid and plasma can smoothen the surface by etching away defects and impurities, which can otherwise initiate cracks under tensile stress. Therefore, as an extension, plasma-assisted solvolysis with HNO_3_ can possibly oxidize and remove non-graphitic or amorphous carbon, potentially promoting a more ordered and graphitic structure in the fibers [28]. In any case, the surface quality and the mechanical properties of the rCFs can be considered excellent and similar those of the vCFs.

## 4. Discussion

The experimental data presented in Section 3 can be further processed to study the Quality and Process Efficiency Aspects, Environmental Aspects, and Cost Aspects of the proposed recycling method. The experimental data were extrapolated to the treatment of 1 kg of CFRCs and are summarized in Table 2, Table 3, Table 4, Table 5 and Table 6. For this projection, some necessary but reasonable assumptions were used. For the process parameters CF retrieval rate, retrieval efficiency, and process time (Table 2), we assumed that the increase in CFRC mass would not affect the experimental values. This is quite reasonable and a modest estimation as in the current experiments, the increase in CFRC mass led to an enhancement in the process efficiency. The total HNO_3_ losses (Table 4) for the dissolution of 1 kg CFRC was calculated from the exponential fit of the experimental results in Figure 4. The solution of the equation for m_o,CFRC_ = 1 kg and m_rCF_ = 0.65 kg results in HNO_3_ losses of 3.20 kg.

These losses were further used to calculate the initial mass and volume of HNO_3_ which are required to dissolve 1 kg of CFRCs. According to the experimental data, about 60% of HNO_3_ is lost during treatment, indicating that processing 1 kg of CFRCs requires about 5.34 kg of HNO_3_ and a volume of about 5.87 L. The plasma power is linearly adjusted for this volume to 1220 W, resulting in a calculated process energy of 6.2 kWh. Moreover, the HNO_3_ loss of 3.20 kg is used for the calculation of the real HNO_3_ wastes after considering that 20% of the stream is trapped in the wet scrubber, yielding a 4 M HNO_3_ solution suitable for CFRC pretreatment. Thus, the final HNO_3_ losses are calculated as 2.56 kg (Table 4). The 1 L volume (0.79 kg, Table 4)) of acetone for cleaning rCFs from 1 kg of CFRCs (~0.65 kg) is realistic and is based on different lab trials. The 1.46 kg of N_2_ gas that was used for plasma ignition was not adjusted and corresponds to the experimental conditions presented above, i.e., an N_2_ gas flow of 4 Lmin^−1^ (N_2_ density 1.165 kg/m^3^). In our scenario, we have assumed that the treatment of 1 kg of CFRCs is feasible by increasing simultaneously the plasma power and HNO_3_ initial volume, without needing to modify any other process parameters. The equipment cost (Table 5) encompasses the purchase, transportation, and installation. The prices are realistic, reflecting a recent system installed in the lab, capable of accommodating approximately 1 kg of CFRCs. The operational costs (Table 6) are adjusted to 1 kg of CFRCs and include the electricity cost, the cost of chemicals (HNO_3_, acetone), and the cost of gas (N_2_). The prices for chemicals and N_2_ are indicative and correspond to large-scale orders, including the transportation costs. Finally, for this scenario, we assumed that the mechanical properties of the retrieved fibers are not affected by the increase in CFRC mass and remain similar to those of the vCFs (Table 3).

The process data calculated above can provide crucial information for the Quality and Process Efficiency Aspects, Environmental Aspects, and Cost Aspects of the recycling process. However, the Circular Economy Aspects and Human Health and Social Impact Aspects cannot be addressed yet as the recycled fibers have not been used in other structures and health/safety analyses of the process have not been finalized. Thus, the complete holistic sustainability assessment cannot be fully implemented yet. Nevertheless, the process data can be used to compare the plasma-enhanced solvolysis process to other already established recycling technologies. Table 7 summarizes important parameters of the most common CFRC recycling processes based on the literature findings.

The data for plasma-enhanced solvolysis are also included, revealing that the main advantages of the process are the quality of fibers and the process capacity. The relatively short treatment times due to the plasma enhancement enable high capacities; thus, a modest (~10 L) low-cost reactor can be used for the treatment of ~1000 tn of CFRCs per year.

The operational costs and the primary energy demand of plasma solvolysis are comparable to those of the other recycling methods. The primary energy demand was calculated according to the recycling content approach proposed in [33]. In this approach, we have assumed that the total composite embodied energy is equal to the energy required for fiber production and the energy for the recycling process is equal to the electrical energy required for the plasma process. However, the main disadvantage of the process is the high environmental impact as it scores the highest GWP among the other recycling methods. This is owed mainly to the use of HNO_3_ and the rather high HNO_3_ losses per treatment. The total GWP value (Table 4) was calculated from the chemical streams and the electrical energy consumed in our process for the treatment of the 1 kg composite. More precisely, the mass flows of chemicals (HNO_3_, H_2_O_2_, acetone, and N_2_) and the electrical energy were multiplied to the corresponding CO_2_ equivalents/kg or kWh of the streams as can be found in [34], and the sum of the products lead to the total GWP value.

As previously discussed, the process is far from being fully optimized. The fine tuning of all process parameters, such as plasma power, plasma configuration and electrodes, reactor materials, gas flow, HNO_3_ volume, and HNO_3_ concentration, is expected to significantly reduce the HNO_3_ losses and, consequently, the environmental impact. Process optimization together with comprehensive Life Cycle Assessment (LCA) and Life Cycle Cost (LCC) analysis are already planned, enabling a holistic evaluation of the process’s sustainability based on the method proposed by the authors.

## 5. Conclusions

In the current work, a novel plasma-enhanced solvolysis process was introduced, aiming at the enhancement of the oxidative degradation of CFRCs. This process synergistically combines the traditional nitric acid solvolysis with plasma chemistry. The experimental results indicate a significant increase in the recovery rate of CFs when plasma is utilized.

A complete process flowchart was presented, highlighting the importance of steps such as solvent regeneration, re-use, and the treatment of wastes for ensuring process sustainability. This study’s findings regarding the impact of composite mass on the plasma-enhanced solvolysis process reveal that the ratio of the recovered CFs’ mass to the HNO_3_ mass lost is a crucial parameter. This ratio influences the process energy consumption, capacity, operational costs, and environmental impact. Using these experimental results along with reasonable assumptions, calculations were performed to assess important parameters, including the efficiency, recovery rates, capacity, fiber quality, energy consumption, raw material usage, operational and installation costs, and environmental impact. These data facilitated a preliminary comparison of the plasma-enhanced solvolysis process with other recycling methods, where the quality of fibers and the process capacity emerged as the main advantages of the technique. However, the environmental impact remains a significant drawback, as the process is associated with the highest GWP due to the use and loss of HNO_3_.

Finally, key sustainability pillars and metrics necessary for a holistic assessment of process sustainability have been proposed. Currently, only the three first pillars can be discussed, limiting the implementation of a fully holistic approach. Further process optimization, along with comprehensive Life Cycle Assessment (LCA) and Life Cycle Cost (LCC) analyses, is underway and will enable a thorough evaluation of the process’s sustainability.

## Figures and Tables

**Table 1:** CFRCs’ initial mass m_o,CFRC_, recovered CFs’ total mass m_rCF_, HNO_3_ total volume loss V_l,HNO3_ (L), process time t, and energy E required for complete dissolution.

No	m_o,CFRC_ (kg)	m_rCF_ (kg)	V_l,HNO3_ (L)	Process Time t (h)	Energy E (kWh)
1 (without plasma)	0.035	0.023	0.200	18	4.52
2	0.037	0.023	0.638	4.5	1.12
3	0.075	0.050	0.655	4.33	1.08
4	0.107	0.070	0.710	5.16	1.29
5	0.163	0.097	0.750	5.33	1.33

**Table 2:** Process data.

Process Parameters	Value	Units
CF retrieval rate	0.13	kg of CFs/(kg of CFRC · h)
CF retrieval efficiency	~100	%
Process time	5	h/(kg of CFRC)
Process electrical energy	6.2	kWh/(kg of CFRC)

**Table 3:** Fiber quality.

Fibers Properties	Value	Units	Comments
Tensile strength	5.3 ± 1.1	GPa	Similar to vCfs
Young modulus	250 ± 10	GPa	Similar to VCFs

**Table 4:** Process wastes, emissions, and Global Warming Potential.

Stream	Value	Units	CO_2_ Equivalents (kg CO_2_ eq per kg Stream) ^a^	Total CO_2_ Equivalents/kg CFRC
HNO_3_	2.56	kg HNO_3_/kg CFRC	1.60	4.10
Acetone	0.79	kg acetone/kg CFRC	2.19	1.73
N_2_	1.46	kg N_2_/kg CFRC	0.43	0.63
H_2_O_2_	Negligible	-	-	-
Electricity	6.2	kWh/(kg of CFRC)	0.21 ^b^	1.32
Total				7.78

^a^ Available online: https://shorturl.at/7XOuM (accessed on 3 October 2024) ^b^ Calculated from the 2023 energy mixture in Greece (~20% coal, 60% natural gas and oil, 20% renewables).

**Table 5:** Infrastructure costs.

Equipment	Value	Units
Plasma reactor—plasma electrodes	3500	€/kg CFRC
Wet scrubbers	500	€/kg CFRC
Plasma generator and electrical monitoring	5000	€/kg CFRC
Pipelines and flowmeters	400	€/kg CFRC
Total	9400	€/kg CFRC

**Table 6:** Operational costs.

Type	Value	Units	Cost	Cost €/kg CFRC
Electricity	6.2	kWh/(kg of CFRC)	0.135 €/KWh ^a^	0.84
HNO_3_	2.56	kg HNO_3_/kg CFRC	1 €/kg	2.56
Acetone	1.00	L acetone/kg CFRC	0.8 €/L	0.80
N_2_	1.25	m^3^/kg CFRc	0.12 €/m^3^	0.18
Total				4.38

^a^ Electricity retail price in Greece, August 2024.

**Table 7:** Young modulus and tensile strength % reduction, capacity, primary energy demand, GWP, and operational costs for different recycling methods.

Process	Young Modulus (GPa)	Tensile Strength (MPa)	Capacity (tn)	Primary Energy Demand (MJ/kg)	GWP (kg CO_2_ eq/kg CFRC)	Cost (Raw, Labor, Process) (€/kg)	Ref.
Mechanical			20	0.27–2.03			[29]
Conventional Pyrolysis	12% reduction	4% reduction	2000	37	2.88	1.2	[27,29,30]
Fluidized Bed Process	5% reduction	18% reduction	100	10	1.54		[27,30]
Chemical Solvolysis	11% reduction	5% reduction		38	1.51	3.6	[29,31,32]
Supercritical Solvolysis	retained	2% reduction	12	20	0.87		[27,29]
Plasma-Enhanced Solvolysis	retained	retained	1000	22	7.78	3.5	

## Data Availability

The original contributions presented in the study are included in the article, further inquiries can be directed to the corresponding author.

## References

[1] Sayam A., Rahman A.N.M.M., Rahman S., Smriti S.A., Ahmed F., Rabbi F., Hossain M., Faruque O. (2022). A review on carbon fber-reinforced hierarchical composites: Mechanical performance, manufacturing process, structural applications and allied challenges. Carbon Lett..

[2] Bledzki A.K., Seidlitz H., Goracy K., Urbaniak M., Rösch J.J. (2021). Recycling of Carbon Fiber Reinforced Composite Polymers—Review—Part 1: Volume of Production, Recycling Technologies, Legislative Aspects. Polymers.

[3] Podara C., Termine S., Modestou M., Semitekolos D., Tsirogiannis C., Karamitrou M., Trompeta A.-F., Milickovic T.K., Charitidis C. (2024). Recent Trends of Recycling and Upcycling of Polymers and Composites: A Comprehensive Review. Recycling.

[4] La Rosa A.D., Banatao D.R., Pastine S.J., Latteri A., Cicala G. (2016). Recycling treatment of carbon fibre/epoxy composites: Materials recovery and characterization and environmental impacts through life cycle assessment. Compos. Part B Eng..

[5] European Council (2020). Directive 200053EC of the European parliament and of the council on end-of life vehicles. Off. J. Eur. Communities.

[6] Shehab E., Meiirbekov A., Amantayeva A., Suleimen A., Tokbolat S., Sarfraz S. (2021). A Cost Modelling System for Recycling Carbon Fiber-Reinforced Composites. Polymers.

[7] Pakdel E., Kashi S., Varley R., Wang X. (2021). Recent progress in recycling carbon fibre reinforced composites and dry carbon fibre wastes. Resour. Conserv. Recycl..

[8] Vincent G.A., de Bruijn T.A., Wijskamp S., Iqbal M., Rasheed A., van Drongelen M., Akkerman R. (2019). Shredding and sieving thermoplastic composite scrap: Method development and analyses of the fibre length distributions. Compos. Part B Eng..

[9] Oliveux G., Dandy L.O., Leeke G.A. (2015). Current status of recycling of fibre reinforced polymers: Review of technologies, reuse and resulting properties. Prog. Mater. Sci..

[10] Meng F., McKechnie J., Turner T., Pickering S.J. (2017). Energy and environmental assessment and reuse of fluidised bed recycled carbon fibres. Compos. Part A Appl. Sci. Manuf..

[11] Okajima I., Sako T. (2017). Recycling of carbon fiber-reinforced plastic using supercritical and subcritical fluids. J. Mater. Cycles Waste Manag..

[12] Kim Y.N., Kim Y.O., Kim S.Y., Park M., Yang B., Kim J., Jung Y.C. (2019). Application of supercritical water for green recycling of epoxy-based carbon fiber reinforced plastic. Compos. Sci. Technol..

[13] Wang Y., Cui X., Ge H., Yang Y., Wang Y., Zhang C., Li J., Deng T., Qin Z., Hou X. (2015). Chemical Recycling of Carbon Fiber Reinforced Epoxy Resin Composites via Selective Cleavage of the Carbon–Nitrogen Bond. ACS Sustain. Chem. Eng..

[14] Yang P., Zhou Q., Yuan X.X., Van Kasteren J.M.N., Wang Y.Z. (2012). Highly efficient solvolysis of epoxy resin using poly(ethylene glycol)/NaOH systems. Polym. Degrad. Stab..

[15] Zhang N., Wu S., Wang C., Cui X., Zhao T., Yuan L., Qi Y., Hou X., Jin H., Deng T. (2022). Efficient catalytic degradation of anhydride-cured epoxy resin by amphiphilic molecule catalysts. Green Chem..

[16] Shi X., Luo C., Lu H., Yu K. (2019). Primary recycling of anhydride-cured engineering epoxy using alcohol solvent. Polym. Eng. Sci..

[17] Zabihi O., Ahmadi M., Liu C., Mahmoodi R., Li Q., Naebe M. (2020). Development of a low cost and green microwave assisted approach towards the circular carbon fibre composites. Compos. Part B Eng..

[18] Sun H., Guo G., Memon S.A., Xu W., Zhang Q., Zhu J.H., Xing F. (2015). Recycling of carbon fibers from carbon fiber reinforced polymer using electrochemical method. Compos. Part A Appl. Sci. Manuf..

[19] Rijo B., Dias A.P.S., Carvalho J.P.S. (2023). Recovery of carbon fibers from aviation epoxy composites by acid solvolysis. Sustain. Mater. Technol..

[20] Hanaoka T., Ikematsu H., Takahashi S., Ito N., Ijuin N., Kawada H., Arao Y., Kubouchi M. (2022). Recovery of carbon fiber from prepreg using nitric acid and evaluation of recycled CFRP. Compos. Part B Eng..

[21] Dang W., Kubouchi M., Yamamoto S., Sembokuya H., Tsuda K. (2002). An approach to chemical recycling of epoxy resin cured with amine using nitric acid. Polymer.

[22] Marinis D., Farsari E., Alexandridou C., Amanatides E., Mataras D. (2024). Chemical recovery of carbon fibers from composites via plasma assisted solvolysis. J. Phys. Conf. Ser..

[23] Das M., Varughese S. (2016). A Novel Sonochemical Approach for Enhanced Recovery of Carbon Fiber from CFRP Waste Using Mild Acid-Peroxide Mixture. ACS Sustain. Chem. Eng..

[24] Bruggeman P.J., Kushner M.J., Locke B.R., Gardeniers J.G., Graham W., Graves D.B., Hofman-Caris R.C.H.M., Maric D., Reid J.P., Ceriani E. (2016). Plasma–liquid interactions: A review and roadmap. Plasma Sources Sci. Technol..

[25] Ashpis D., Laun M., Griebeler E. (2017). Progress toward Accurate Measurements of Power Consumption of DBD Plasma Actuators. AIAA J..

[26] (2020). Standard Test Method for Tensile Strength and Young’s Modulus of Fibers.

[27] Markatos D.N., Katsiropoulos C.V., Tserpes K.I., Pantelakis S.G. (2021). A holistic End-of-Life (EoL) Index for the quantitative impact assessment of CFRP waste recycling techniques. Manuf. Rev..

[28] Pitto M., Fiedler H., Kim N.K., Johannes C., Verbeek R., Allen T.D., Bickerton S. (2024). Carbon fibre surface modification by plasma for enhanced polymeric composite performance: A review. Compos. Part A Appl. Sci. Manuf..

[29] Meng F., Olivetti E.A., Zhao Y., Chang J.C., Pickering S.J., McKechnie J. (2018). Comparing Life Cycle Energy and Global Warming Potential of Carbon Fiber Composite Recycling Technologies and Waste Management Options. ACS Sustain. Chem. Eng..

[30] Qureshi J. (2022). A Review of Recycling Methods for Fibre Reinforced Polymer Composites. Sustainability.

[31] Krauklis A.E., Karl C.W., Gagani A.I., Jørgensen J.K. (2021). Composite Material Recycling Technology—State-of-the-Art and Sustainable Development for the 2020s. J. Compos. Sci..

[32] Ateeq M. (2023). A state of art review on recycling and remanufacturing of the carbon fiber from carbon fiber polymer composite. Compos. Part C Open Access.

[33] Borda F., Ingarao G., Ambrogio G., Gagliardi F. (2024). Cumulative energy demand analysis in the current manufacturing and end-of-life strategies for a polymeric composite at different fibre-matrix combinations. J. Clean. Prod..

[34] https://shorturl.at/7XOuM.

